# Supplementary material for: HIVprotI: an integrated web based platform for prediction and design of HIV proteins inhibitors
Source: J Cheminform. 2018 Mar 9;10:12. doi: 10.1186/s13321-018-0266-y (PMC5845081; doi:10.1186/s13321-018-0266-y)
Supplement: Supplementary file 2 — Additional file 2. Source code of HIVProtI web server. [file 13321_2018_266_MOESM2_ESM.zip › HIVprotI_Source-code/hivprotI/weka-3-6-6/doc/weka/estimators/ConditionalEstimator.html]

ConditionalEstimator


---


|  |  |  |
| --- | --- | --- |
| **Overview** \| **Package** \| **Class** \| **Tree** \| **Deprecated** \| **Index** \| **Help** \| **Weka's home** | |  |
| **PREV CLASS**   **NEXT CLASS** | **FRAMES**    **NO FRAMES**     **All Classes** |  |
| SUMMARY: NESTED \| FIELD \| CONSTR \| METHOD | DETAIL: FIELD \| CONSTR \| METHOD |  |


---


## weka.estimators Interface ConditionalEstimator

**All Superinterfaces:**: RevisionHandler

**All Known Implementing Classes:**: DDConditionalEstimator, DKConditionalEstimator, DNConditionalEstimator, KDConditionalEstimator, KKConditionalEstimator, NDConditionalEstimator, NNConditionalEstimator

---

``` public interface ConditionalEstimator extends RevisionHandler ```

Interface for conditional probability estimators. Example code:

```` ```
   NNConditionalEstimator newEst = new NNConditionalEstimator();

   // Create 50 random points and add them
   Random r = new Random(seed);
   for(int i = 0; i < 50; i++) {
     int x = Math.abs(r.nextInt() % 100);
     int y = Math.abs(r.nextInt() % 100);
     System.out.println("# " + x + "  " + y);
     newEst.addValue(x, y, 1);
   }

   // Pick a random conditional value
   int cond = Math.abs(r.nextInt() % 100);
   System.out.println("## Conditional = " + cond);

   // Print the probabilities conditional on that value
   Estimator result = newEst.getEstimator(cond);
   for(int i = 0; i <= 100; i+= 5) {
     System.out.println(" " + i + "  " + result.getProbability(i));
   }
``` ```` 

**Version:**
:   $Revision: 1.7 $

**Author:**
:   Len Trigg (trigg@cs.waikato.ac.nz)

---

| **Method Summary** | |
| --- | --- |
| `void` | `addValue(double data, double given, double weight)`             Add a new data value to the current estimator. |
| `Estimator` | `getEstimator(double given)`             Get a probability estimator for a value |
| `double` | `getProbability(double data, double given)`             Get a probability for a value conditional on another value |

**Methods inherited from interface weka.core.RevisionHandler**

`getRevision`

**Method Detail**

### addValue

```
void addValue(double data,
              double given,
              double weight)
```

:   Add a new data value to the current estimator.

    :   **Parameters:**: `data` - the new data value: `given` - the new value that data is conditional upon: `weight` - the weight assigned to the data value

---


### getEstimator

```
Estimator getEstimator(double given)
```

:   Get a probability estimator for a value

    :   **Parameters:**: `given` - the new value that data is conditional upon **Returns:**: the estimator for the supplied value given the condition

---


### getProbability

```
double getProbability(double data,
                      double given)
```

:   Get a probability for a value conditional on another value

    :   **Parameters:**: `data` - the value to estimate the probability of: `given` - the new value that data is conditional upon **Returns:**: the estimator for the supplied value given the condition


---


|  |  |  |
| --- | --- | --- |
| **Overview** \| **Package** \| **Class** \| **Tree** \| **Deprecated** \| **Index** \| **Help** \| **Weka's home** | |  |
| **PREV CLASS**   **NEXT CLASS** | **FRAMES**    **NO FRAMES**     **All Classes** |  |
| SUMMARY: NESTED \| FIELD \| CONSTR \| METHOD | DETAIL: FIELD \| CONSTR \| METHOD |  |


---
